# A Small β-Carboline Derivative “B-9-3” Modulates TGF-β Signaling Pathway Causing Tumor Regression *in Vivo*

**DOI:** 10.3389/fphar.2018.00788

**Published:** 2018-07-19

**Authors:** Hui Zhong, Abdelkader Daoud, Jichun Han, Xiaohong An, Caili Qiao, Lanlan Duan, Yichuan Wang, Zhenfeng Chen, Jia Zhou, Jing Shang

**Affiliations:** ^1^State Key Laboratory of Natural Medicines, Jiangsu Key Laboratory of TCM Evaluation and Translational Research, School of Traditional Chinese Pharmacy, China Pharmaceutical University, Nanjing, China; ^2^State Key Laboratory for Chemistry and Molecular Engineering of Medicinal Resources, School of Chemistry and Pharmacy, Guangxi Normal University, Guilin, China

**Keywords:** β-carboline derivative, TGF-β signaling pathway, tumor micro-environment, antitumor, immunomodulatory effects

## Abstract

Targeting tumor microenvironment (TME) is crucial in order to overcome the anti-cancer therapy resistance. In this study, we report the antitumor activity of a newly synthesized β-carboline derivative “B-9-3.” Here, this small molecule showed a promising antitumor activity *in vivo* along with an enhanced immune response as reflected by a reduction of regulatory T cells and increased CD4+/CD8+ T cells. Further, B-9-3 decreased the number of myofibroblasts not only in the tumor but also in the lung suggesting an anti-metastatic action. The reduction of myofibroblasts was associated with lower expression of epithelial-to-mesenchymal transition markers and a decrease of phosphorylated SMAD2/3 complex indicating the implication of TGF-β signaling pathway in B-9-3’s effect. The blockade of myofibroblasts induction by B-9-3 was also verified *in vitro* in human fibroblasts treated with TGF-β. To elucidate the mechanism of B-9-3’s action on TGF-β pathway, first, we investigated the molecular interaction between B-9-3 and TGF-β receptors using docking method. Data showed a weak interaction of B-9-3 with the ATP-binding pocket of TGFβRI but a strong one with a ternary complex formed of extracellular domains of TGFβRI, TGFβRII, and TGF-β. In addition, the role of TGFβRI and TGFβRII in B-9-3’s activity was explored *in vitro*. B-9-3 did not decrease any of the two receptors’ protein level and only reduced phosphorylated SMAD2/3 suggesting that its effect was more probably due to its interaction with the ternary complex rather than decreasing the expression of TGF-β receptors or interfering with their ATP-binding domains. B-9-3 is a small active molecule which acts on the TGF-β signaling pathway and improves the TME to inhibit the proliferation and the metastasis of the tumor with the potential for clinical application.

## Introduction

Cancer appears as one of the leading causes of death worldwide, accounting for 8.2 million deaths in (World Health Organization, 2012). In general, three types of cancer are more prevalent among all malignancies including lung, breast, and colon. Interestingly, tumor microenvironment (TME) of these cancers is recognized as a critical participant in tumor progression and therapeutic responses (Graves et al., 2010; Beauchemin, 2011; Place et al., 2011). In fact, recent evidence attributes successive failures of therapies to, not only drug targets mutation but also TME and tumor-associated immunosuppression. It has been proven that the TME of lung, breast and colon cancers has an active inflammatory characteristic and plays many roles in tumor progression and metastasis by favoring the epithelial-to-mesenchymal transition (EMT) leading to cancer initiation, progression, and metastasis (Heinrich et al., 2012). EMT is the process by which epithelial cells acquire the mesenchymal trait resulting in enhanced tumor cell motility and invasiveness. Cells undergoing EMT have reduced epithelial markers such as E-cadherin and cytokeratin, and up-regulated mesenchymal markers like vimentin and *N*-cadherin (Yu et al., 2014). It is well established that EMT is under control of TGF-β signaling pathway (Palena et al., 2012). Interestingly, TGF-β pathway is also a key inducer of cancer-associated fibroblasts (CAFs) in the microenvironment. CAFs or known as activated fibroblasts are mainly formed from fibroblasts through a phenomenon called “fibroblast to myofibroblast transition" (FMT). Due to the higher CAFs proliferative activity, it persistently exists in the tumor milieu, and cannot be removed by apoptosis. Growing evidence also suggests that CAFs are derived from malignant epithelial cells via EMT (Zhang and Liu, 2013; Yoshida et al., 2014). EMT plays an essential role in cancer metastasis and resistance to chemotherapy, radiation and even specific small-molecule-targeted therapies (Xie et al., 2014).

From a therapeutic aspect, the need for novel compounds to fight the evasion of tumors is a necessity as the disease remains a significant threat challenging human life. Naturally occurring phytochemicals from various plants are always an essential source in the discovery of new therapeutic agents. Here we investigate the antitumor and immunomodulatory effects of a newly synthesized β-carboline derivative B-9-3 in two animal models of lung cancer, and we elucidate its mechanism of action with an emphasis on the implication of TGF-β signaling pathway in this action. The unique mechanisms of B-9-3 also brings a new conception to design the active antitumor molecules which acts on the TGF-β signaling pathway.

## Materials and Methods

### Reagents

#### B-9-3

B-9-3 was synthesized by “Xinjiang Huashidan Pharmaceutical Research CO., LTD.” (Urumqi, China) among other β-carboline derivatives that have already been investigated elsewhere (Monsef et al., 2004; Li et al., 2007; Han et al., 2012). The chemical structure and characterization of B-9-3 are indicated in the Section “Results” of this chapter. For *in-vivo* studies, B-9-3 was dissolved in sterile saline and was given intraperitoneally (i.p) at three doses: 1, 10 and 20 mg/kg, three times a week for 3 weeks.

### Cell Culture

#### A549 Cell Line

The cell line is a human non*-*small cell lung carcinoma (NSCLC) cell line that is widely used to establish human lung cancers. Models using this cell line are often developed in female BALB/c nude mice (Shi et al., 2013). A549 cell line was maintained in RPMI-1640 medium (Life Technologies, Inc., CA, United States). Supplemented with 10% (v/v) heat-inactivated fetal bovine serum, 2 mM glutamine,10 mM HEPES buffer [4-(2-hydroxyethyl)-1-piperazineethanesulfonic acid], 100 U/ml streptomycin and 100 U/ml penicillin at 37°C in a humid atmosphere (5% CO_2_, 95% air). For subculture, cells were detached with 0.25% trypsin-EDTA (Ethylene Diamine Tetraacetic Acid) solution, and 1 × 10^6^ cells were seeded into new flasks.

#### Lewis Lung Cancer (LLC) Cell Line

The LLC cell line was kindly provided by Prof. Guo QingLong from China Pharmaceutical University. These LLC cells line are being established initially from male C57BL mice and are widely used to study lung metastases and mechanism of action of different chemotherapeutic agents (Bertram and Janik, 1980; Isakov et al., 1984; Kraus-Berthier et al., 2000). In our study, we selected this model because we wanted to explore the immune system response about the drug. Cells were maintained in RPMI-1640 medium (Life Technologies, Inc., CA, United States). Supplemented with 10% (v/v) heat-inactivated fetal bovine serum, 2 mM glutamine, 10 Mm HEPES buffer, 100 U/ml streptomycin and 100 U/ml penicillin at 37°C in a humid atmosphere (5% CO_2_, 95% air). For subculture, cells were detached with 0.25% trypsin-EDTA solution, and 1 × 10^6^ cells were seeded into new flasks.

#### Human Fibroblast

Human fibroblasts were extracted from colleagues at China Pharmaceutical University. Cells were maintained in Dulbecco’s Modified Eagle’s Medium (DMEM) supplemented with 10% (v/v) heat-inactivated fetal bovine serum, 2 mM glutamine, 10 mM HEPES buffer, 100 U/ml streptomycin and 100 U/ml penicillin at 37°C in a humid atmosphere (5% CO_2_, 95% air). For subculture, cells were detached with 0.25% trypsin-EDTA solution, and 1 × 10^6^ cells were seeded into new flasks.

#### Immunofluorescence of FSP-1 and α-SMA

Human fibroblasts were cultured in DMEM supplemented with 10% (v/v) fetal bovine serum at 37°C and 5% CO_2_ for 24 h. Then with DMEM supplemented with 0.1% BSA and in the same medium supplemented with TGF-β1 (5 ng/ml) for 5 days as previously mentioned (Calone and Souchelnytskyi, 2012). Cells were fixed in 3.7% paraformaldehyde, permeated in 0.1% Triton X-100, blocked with 2% BSA and incubated with rabbit monoclonal antibody against human α-SMA and mouse monoclonal antibody against human FSP-1 at 4°C for 24 h. Cells were washed and incubated with secondary antibodies (goat anti-mouse TRITC antibody and goat anti-rabbit FITC antibody) for 1 h at 37°C in the dark. Cells were and counterstained with DAPI using ProLong^^®^^Acv Gold Antifade Mountant with DAPI (Invitrogen cat# P-36931). Pictures were viewed using Olympus FLUOVIEW Viewer software (Tokyo, Japan).

#### Gene Silencing of TGF-β Receptor 2 (TGFβRII)

Human fibroblasts were cultured in DMEM supplemented with 10% (v/v) fetal bovine serum at 37°C and 5% CO_2_ until they reach 90% of confluence. Cells were harvested using 0.25% trypsin-EDTA and resuspended in fresh serum-containing media, after centrifugation. Validated TGFβRII siRNA (Invitrogen, cat#AM51331) and the negative control siRNA (Invitrogen, cat#AM4611) stock solutions were prepared in RNAase-free water and kept on ice until use (or at −20°C). For transfecting TGFβRII into fibroblasts, the reverse protocol was used. In each well to be transfected, 30 pmol RNAi duplex (siRNA) was prepared in 500 μl Opti-MEM^^®^^ I Medium (Invitrogen, cat# 31985-062) without serum and added in each well of six-well plate. Then, 5 μl of Lipofectamine^^®^^ RNAiMAX (Invitrogen, cat#13778-075) was introduced to each well containing the diluted RNAi molecules, and the mixture was incubated for 10–20 min at room temperature. Cells were diluted in complete growth medium without antibiotics so that 2.5 ml contains the appropriate number of cells to give 30–50% confluence 24 h after plating (between 20,000 and 50,000 cells/well). Then, to each well with RNAi duplex-Lipofectamine ^^®^^RNAiMAX complexes, 2.5 ml of the diluted cells were added, and cells were incubated for the indicated time.

### Animal Models

#### Human A549 Lung Cancer Model

Female BALB/c nude mice, aged 35–40 days and weighted 18–22 g, were supplied from Shanghai SLAC animal laboratory, under a license number: SCXK (Shanghai) 2007-0005. Animals were housed in a room with constant temperature (23°C) and 12-h light/dark cycle with free access to standard mouse chow and water. Mice were kept in sterile conditions for 10 days of environmental adaptation. On the day of the injection, tumor cells were harvested, and cell viability was determined by trypan blue dye exclusion on a Countess^TM^ automated cell counter (Life technologies, Invitrogen) and was greater than 95%.

For the orthotopic xenograft, mice were anesthetized using an 8% chloral hydrate solution (1 ml/20 g), and a skin incision was made on the left chest. The muscles were separated to expose the ribs and human lung cancer cells A549 were injected into the intercostal space on the right lung; finally, the skin was sutured. The total number of cells was 2 × 10^6^ in 200 μl saline as previously mentioned (Mathieu et al., 2004). Two weeks later, when tumors were formed, animals were randomly divided into groups of six animals per each (except the control group that had 12 mice). Mice in all groups were sacrificed 21 days after treatment. All experiments were done according to the Guides for the Care and Use of Laboratory Animals and approved by China Pharmaceutical University Animal Care Committee.

#### Mouse LLC Lung Cancer Model

Male C57BL/6J mice were purchased at 6–8 weeks of age from Nanjing JunKe Biotech Animal laboratory (Nanjing, China) under a license number: SCXK (Shanghai) 2007-004. Animals were housed in a room with constant temperature (23°C) and 12-h light/dark cycle with free access to standard mouse chow and water. Mice were kept in sterile conditions for 10 days of environmental adaptation. On the day of the injection, tumor cells were harvested, and cell viability was determined by trypan blue dye exclusion on a Countess^TM^ automated cell counter (Life technologies, Invitrogen) and was greater than 95%. Mice were injected subcutaneously with 1 × 10^6^ LLC cells in 100 μl saline in the right flank (DuPage et al., 2009). After two successive *in-vivo* passaging in C57BL mice, finally tumors were extracted, and resulted cells were injected in the study group. Four days later, mice were randomly divided into five groups (*n* = 10 animals/group): Saline group; Taxol treated group, B-9-3 high dose group, B-9-3 medium dose group and B-9-3 low dose group. Drugs’ schedules were as mentioned above (“Materials and methods”). Tumor width (*W*) and length (*L*) were measured every other day by calipers. The tumor volume was calculated according to the following formula: Tumor volume = 0.52 × L × W^2^, where *L* is the length and *W* is the width of the tumors. Mice in all groups were sacrificed 21 days after treatment. All experiments were done according to the Guides for the Care and Use of Laboratory Animals and approved by China Pharmaceutical University Animal Care Committee.

#### Histopathological Analysis

Hematoxylin and eosin (H&E) staining were performed on heart, liver, lung, kidney, spleen, thymus, and tumor. Tissues were collected after sacrificing animals and were fixed in 10% paraformaldehyde solution. Sections of 3–5 μm were made from paraffin-embedded tissues, and the staining was performed at Jiangsu provincial integrated Chinese*-*western medicine hospital.

#### Flow Cytometry Analysis

This analysis for circulating CD4^+^, CD8^+^, and CD4^+^CD25^+^FoxP_3_ was performed on both fresh blood and spleen using a kit (eBioscience, CA, United States) following the manufacturer’s protocol. Briefly, just after animals were sacrificed, new blood was collected in small tubes filled with 1/10 v/v EDTA-2Na to stop coagulation. For CD4^+^, CD8^+^ T cells, to a 100 μl of each sample, 1 μl of anti-mouse CD4-FITC and 1 μl of anti-mouse CD8-PE were added, and the mixtures were kept in the dark at 4°C for 20 min. Lysis buffer was then added, and the samples were further incubated in the dark at 4°C for 10 min. The mixtures were centrifuged at 1500 rpm for 3–5 min and washed with phosphate buffer saline (PBS) once. Finally, 200 μl of PBS was added to each sample, and FACS analysis was carried out using a FACSCalibur flow cytometer. For CD4^+^CD25^+^FoxP_3_ T cells, 1 μl of anti-mouse CD4-FITC and 1 μl of anti-mouse CD25-PE were added, and the mixtures were kept in the dark at 4°C for 30 min. After washing cells in cold flow cytometry staining buffer, the pellet was centrifuged, and the supernatant was discarded. Resuspended cells in freshly prepared fixation/permeabilization working solution were again incubated at 4°C for between 30 min and 18 h in the dark. Another series of washing was performed, and cells were stained with anti-mouse Foxp3-PE-Cy5 (FJK-16s) antibody at 4°C for at least 30 min in the dark. Samples were again washed and resuspended in an appropriate volume of flow cytometry staining buffer and analyzed on a flow cytometer.

For analyzing spleen’s lymphocytes, tissues were placed in a small beaker containing 1–2 ml of PBS and were cut extensively until a homogeneous liquid was formed which was treated as fresh blood.

#### Immunohistochemistry

Sections of 5 μm in thickness paraffin block were placed on adhesive-coated slides. Then, sections were deparaffinized with xylene, rehydrated in graded ethanol (from 100 to 70%) and heated for 30 min in a sodium citrate buffer to increase epitope exposure. Additionally, slides were treated with 0.3% (v/v) H_2_O_2_ in for 5 min, washed with 0.01 M PBS and blocked with 1% BSA, 0.2% Tween 20 in PBS for 1 h at room temperature. Slides were then incubated overnight at 4°C with monoclonal antibody against: Forkhead box P3 (Foxp3 CellSignaling, Danvers, MA, United States), monoclonal antibody against fibroblast surface protein 1, FSP-1 (Abcam, Cambridge, United Kingdom), monoclonal antibody against alpha-smooth muscle actin, α-SMA (Abcam, Cambridge, United Kingdom), and monoclonal antibodies against E-cadherin and *N*-cadherin (CellSignaling, Danvers, MA, United States). The optimal dilution for all antibodies was 1:100. The reaction antigen-antibody was visualized with avidin-biotin-peroxidase by using 3,3-diaminobenzidine as the chromogen which resulted in brown staining. Slides were counterstained in Harris hematoxylin. Samples were then dehydrated, preserved with a coverslip and reviewed using light microscopy. Pictures were viewed using Olympus FLUOVIEW Viewer software (Tokyo, Japan).

#### qPCR Analysis

Total mRNA from animal tissues was isolated using TRIzol Reagent (Invitrogen). RNA was reverse-transcribed using The PrimeScript RT Master Mix (Perfect Real Time) (Takara, Dalian, China). Real-time quantitative RT-PCR was performed using the iCycler 5 thermal cycler (BioRad, CA, United States). Each cDNA was amplified in a 20 μl volume using the SYBR Premix EX Taq kit (Takara, Dalian, China), with a 500 nM final concentration of each primer. The amplification specificity was checked using melting curve analysis. For each cDNA, all target gene mRNA levels were normalized to GAPDH mRNA levels. Results are expressed as the ratio of normalized target gene mRNA levels in treated groups relative to those in the saline group.

#### Mouse TGF-β1 ELISA Analysis

The concentrations of TGF-β1 in the serum were determined by ELISA kits (eBioscience, CA, United States) according to manufacturer’s protocols. Briefly, serum samples were diluted 1/5 in PBS and were acidified by a 1 N solution of HCl to activate latent TGF-β1 to the immunoreactive form. The samples were then neutralized using a 1 N solution of NaOH. Activated samples and standard curve dilutions were added into a 96 well plate and were allowed to react with the capture antibody overnight at 4°C. After a series of aspiration/washing, a detection antibody diluted was added, and the plate was incubated at room temperature for 1 h. The wells were again washed, and the enzyme Avidin-HRP was added. After incubation at room temperature for 30 min, the plate was cleaned, and a substrate solution was introduced into each well. The reaction was stopped 15 min later using a stop solution (2N H_2_SO_4_), and absorbance was read at 450 nm. Samples concentrations were determined using the equation obtained from the standard curve. Data were represented as Mean ± SD (standard of deviation).

#### Western Blot Analysis

Western blotting was conducted on total protein from tumors. One hundred milligrams of tissue was broken using a protein lysis buffer following the manufacturer’s instructions (ApplyGene, Beijing, China). After determining the protein samples’ concentration, a Laemmli loading buffer containing 100 mM of dithiothreitol (DTT), was added at a ratio 4:1 (sample: loading buffer). Samples were then kept at −70°C for extended term storage or at −20°C for short time storage. For western blotting, equal amounts (40 μg) of proteins were then fractionated on 12% SDS-PAGE in the Mini-PROTEAN II system (BioRad, Hercules, CA, United States). The running was performed in two phases (S1) with a voltage of 80 V and time of 30 min and (S2) of 120 V and 1 h of time. After determining the localization of the protein of interest, the gel was cut and then transferred onto a nitrocellulose paper for 45 min at 10 V. Membranes were then blocked in 2% BSA solution (0.4 g of BSA in 20 ml of TBST) for 1 h 30 min. After few times washing, the nitrocellulose paper was incubated with different first antibodies overnight at 4°C. The list of first antibodies includes: mouse monoclonal anti-β-actin (Cell Signaling cat#3700), rabbit monoclonal anti-FoxP3 (Cell Signaling cat#12653), rabbit monoclonal anti-SMAD2/3 (Cell Signaling cat#8685), rabbit monoclonal anti-Phospho-SMAD2/3 (Cell Signaling cat#8828), rabbit monoclonal anti-E-Cadherin (Cell Signaling cat#3195), rabbit monoclonal anti-*N*-Cadherin (Cell Signaling cat#13116), rabbit monoclonal anti-α-smooth muscle actin (α-SMA) antibody (Abcam cat#ab5694), mouse monoclonal anti-fibroblast surface protein 1 (FSP-1) antibody (Abcam cat#ab11333), rabbit monoclonal anti-TGFβRI (Cell Signaling cat#3712) and rabbit polyclonal anti-TGFβRII (Abcam cat#ab61213). After overnight incubation, the membranes were washed several times and incubated with the corresponding secondary antibodies: goat anti-mouse (Cell Signaling cat#7076) and goat anti-rabbit (Cell Signaling cat#7074), monoclonal antibodies for 2 h. The samples were again washed in TBST three times and incubated with a developing solution (a mixture of 1/1of Sigma developing solutions “WestPico”: 0.5 ml/sample). Finally, the paper was dried, and the blots were analyzed with Quantity One software (BioRad, CA, United States). All proteins were normalized against β-actin.

#### Docking of TGF-β Receptors and B-9-3

A common technique central to receptor-ligand interactions is docking. Several docking methods are available; we used CDOCKER which uses a CHARMm-based molecular dynamics (MD) scheme to dock ligands into a receptor binding site. Random ligand conformations are generated. The conformations are then translated into the binding site. A final minimization is then used to refine the ligand poses. Minimized ligands are directly used. In this step, TGFβ receptor I or the ternary complex of (TGF-β1, TGFβRI, and TGFβRII) were held rigid while the drug was allowed to flex during the refinement. Meanwhile, the CHARMm was used to perform a simulation on the TGF-β receptors–B-9-3 complex. The receptors’ active sites were located before ligand docking. Random orientations of the confirmations were produced by translating the center of the ligand to a specific location within the receptor active site and performing a series of random orientations. This process continues until either the desired number of low-energy orientations were found, or the maximum numbers of wrong orientations have been tried. For each final pose, the CHARMm energy (interactions energy plus ligand strain) and the interaction energy alone were calculated. The poses were sorted by CHARMm energy and the top scoring (most negative, thus favorable to binding) poses were retained. For docking studies, the result of a docking run was expressed as a set of poses which allowed us to identify hydrophobic interactions or hydrogen bonds at varying degrees of detail. All figures and all subsequent ribbon drawings are prepared using “Accelrys Discovery studio” molecular graphics system.

#### Statistical Analysis

Data are represented as Mean ± SD (standard of deviation). A *T*-test was used for statistical analysis between two groups. Significant differences were accepted when *P* < 0.05. A *P* value ≤ 0.05 was considered significant (^∗^ or ^#^*p* ≤ 0.05, ^∗∗^ or ^##^*P* ≤ 0.01, ^∗∗∗^ or ^###^*P* ≤ 0.001).

## Results

### Toxicity Evaluation

**FIGURE 1:**
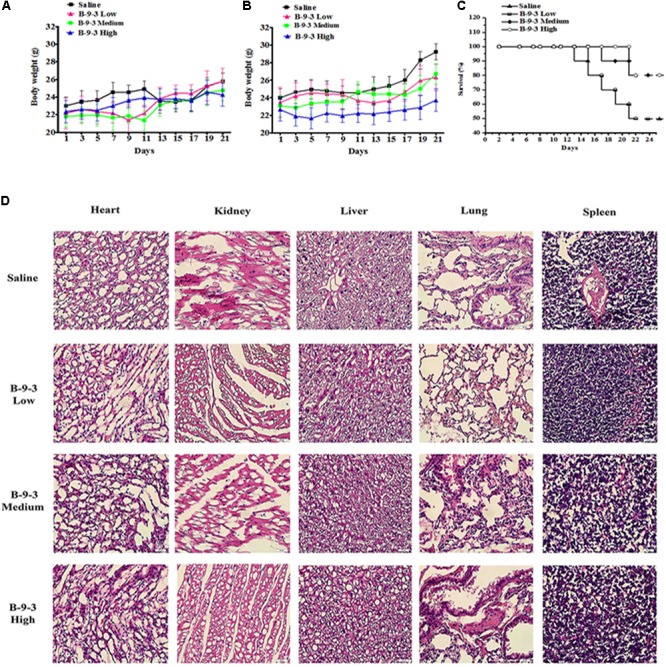
Toxicity evaluation of B-9-3. **(A)** Body weight assessment in A549 model; **(B)** Body weight assessment in LLC model; **(C)** Survival rate in LLC model; **(D)** H&E staining of tissues from LLC model. C57BL mice were injected with 10^6^ cells in the right flank and mice were killed on day 22. B-9-3 was dissolved in sterile saline and was given intraperitoneally (i.p) at three doses: 1, 10, and 20 mg/kg, three times a week for 3 weeks.

Indexes of drug toxicity such as overall survival (OS) rate, weight loss was continuously observed during the whole treatment. **Figures 1A,B** show the body weight assessment in mice from both models. In A549 model, the body weight of saline group continued to increase in during the experiment because the tumor growth and went from 20.3 g at the beginning to 22.2 g in the end. While B-9-3 at both low and high dose caused a slight decrease of body weight (a reduction of 0.9 g for low dose and 0.5 g for high dose), medium dose enhanced the animals’ weight (0.9 g). However, there was no overall significant difference between the body weight of the B-9-3-treated groups and that of the saline group. In LLC model, Tumor-bearing control mice (saline) had higher body weights that stabilized in the middle of the study; however, by the end of the experiment, they increased significantly compared to normal mice due to tumors (about 29 g in the end). Mice treated with B-9-3 at low and medium doses had increased body weights during the study and reached almost 26 g in the end. Meanwhile, the body weights of mice receiving B-9-3 at high dose decreased during the first week of treatment from 22 to 21 g then went back to the initial value of 22 g and stabilized during the long part the experiment until it increased to 23 g in the end.

The OS in LLC model was determined using Kaplan–Meier curve (**Figure 1C**). The saline group had the least survival rate of 50% at the end of the study. While the low dose of B-9-3 did not show significant enhancement of animal survival (=50% in the period), both medium and high doses of B-9-3 showed better results in term of OS as 80% of the mice were still alive by the end of the experiment.

To further verify the absence of toxic effect of B-9-3 in LLC model, the tissues of heart, liver, lung, kidney, and spleen were fixed in 10% neutral buffered paraformaldehyde solution and embedded in paraffin. Sections of 3–5 μm were stained with H&E microscopic evaluation and examined by a pathologist. **Figure 1D** shows the histopathological staining performed in different organs at the end of the experiment. The histopathological staining showed no cytotoxic effect of B-9-3 at any dose on any organ.

### *In Vivo* Antitumor Efficacy

**FIGURE 2:**
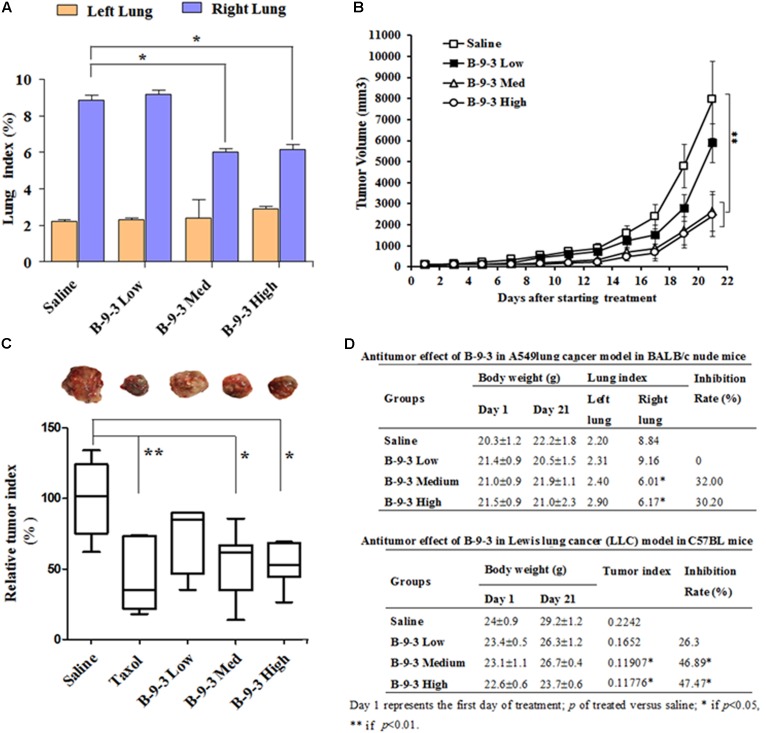
Determination of the *in vivo* antitumor efficacy of B-9-3. **(A)** Lung index assessment in BALB nude mice injected with human lung cancer cells A549; **(B)** Tumor volume assessment in C57BL mice injected with LLC cells; **(C)** Assessment of tumor index in C57BL mice injected with LLC cells; **(D)** Comparison of the antitumor effect of B-9-3 in BALB nude mice and C57BL immunocompetent mice. Female BALB nude mice were orthotopically injected with human lung cancer cells A549 on the right lung; the total number of cells was 1 × 10^6^ in 200 μl saline; 2 weeks later, when tumors were formed, animals were randomly divided into groups of six animals per each and were treated as described in “Materials and methods” section. C57BL mice were injected with 10^6^ cells in the right flank; after 4 days; different drugs were given i.v or i.p for 3 weeks, following the schedule for each drug.

To assess the *in vivo* anti-cancer efficacy of B-9-3 in the studied models, tumor volume in each group was monitored every other day. In A549 model, lungs were weighed at the end of the experiments, and the tissue index was calculated. **Figures 2A,D** demonstrate that B-9-3 significantly decreased the tumor growth, with 32% inhibition at medium dose and 30.2% inhibition at the high dose. Meanwhile, low dose did not show any anti-tumor potential effect. In LLC model and as demonstrated in **Figure 2B**, tumors of saline group expanded gradually at a limited rate in the beginning; however, after 14 days post-therapy the speed of tumor growth increased, and the volume reached about 7900 mm^3^ by the end of the experiment. B-9-3 at low dose did not reduce the tumor growth in a significant way. However, both medium and high dose of B-9-3 decreased the tumor burden significantly, and the volumes reached only about 2600 mm^3^ for medium dose and about 2400 mm^3^ for high dose, on the last day. Also, after all, animals were sacrificed on day 22, tumors were collected and weighed, and the relative tumor index from each group is expressed in **Figures 2C,D**. Low dose B-9-3 did not decrease the tumor volume, and it had the second biggest tumor index, after saline group. The groups treated with medium and high doses of B-9-3 had smaller tumors in comparison to the saline group. **Figure 2D** showed the inhibitory rate of B-9-3 reached about 47.5% at the high dose.

### The Immunomodulatory Effect of B-9-3

Because the antitumor inhibition by B-9-3 in immunocompetent mice was higher than that in nude mice we speculated that B-9-3 might possess immunostimulatory activity. Therefore, we measured the percentages of CD4^+^ and CD8^+^ T cells in peripheral blood and spleens of all animals. In this step, four groups of tumor-free mice were added and included: Normal group, normal mice receiving low dose B-9-3, normal mice receiving medium dose B-9-3 and normal mice receiving B-9-3 at the high dose.

**FIGURE 3:**
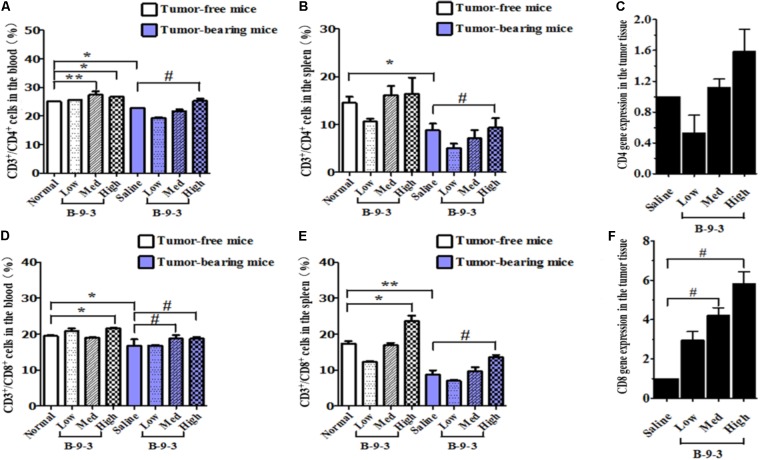
Effect of B-9-3 on CD3^+^/CD4^+^ and CD3^+^/CD8^+^ T cells. **(A)** CD3^+^/CD4^+^ cells in the blood; **(B)** CD3^+^/CD4^+^ cells in spleen; **(C)** CD4 gene expression in the tumor tissue; **(D)** CD3^+^/CD8^+^ cells in the blood; **(E)** CD3^+^/CD8^+^ cells in the spleen; **(F)** CD8 gene expression in the tumor tissue. C57BL mice were injected with 10^6^ cells in the right flank and mice were killed on day 22, fresh blood was collected in EDTA tube, and flow cytometry analysis was performed as described previously. “^∗^” versus normal group and “^#^” versus saline group; ^∗^ or ^#^ if *p* < 0.05 and ^∗∗^ or ^##^ if *p* < 0.01.

It is well documented that the reduction of CD4^+^ and CD8^+^ populations occurred in the peripheral blood of patients suffering from different cancers. A similar observation was also obtained from animal studies (Rita and Young, 2004; Hadrup et al., 2013). Here, we performed flow cytometry to analyze the level of CD4^+^ and CD8^+^ T cells in both the blood and the spleen. First and as shown in **Figure 3**, cancer mice had low levels of both CD4^+^ and CD8^+^ T cells in both the circulating fluid compartment (blood) and the spleen (Saline versus Normal). Additionally, **Figure 3** also showed that B-9-3, in both standard and tumor-bearing mice, increased the level of CD4^+^ and CD8^+^ T cells in both the blood and the spleen which may explain the higher antitumor effect of this drug in immunocompetent mice compared to nude mice. For the verification, whether the increase of circulatory and splenic CD4^+^/CD8^+^ T cells influenced their number in the TME, tumor tissues were collected, and total RNA was extracted as mentioned above. qPCR was then conducted to assess the mRNA level of CD4 and CD8 markers. **Figure 3C** shows that the mRNA levels of CD4 in the group treated with B-9-3 at both medium and high doses were higher than that of saline group. This increase of CD4 expression was dose-dependent. Further, CD8 cells were also found significantly and dose-dependently enhanced in B-9-3 treated mice as revealed in **Figure 3F**.

**FIGURE 4:**
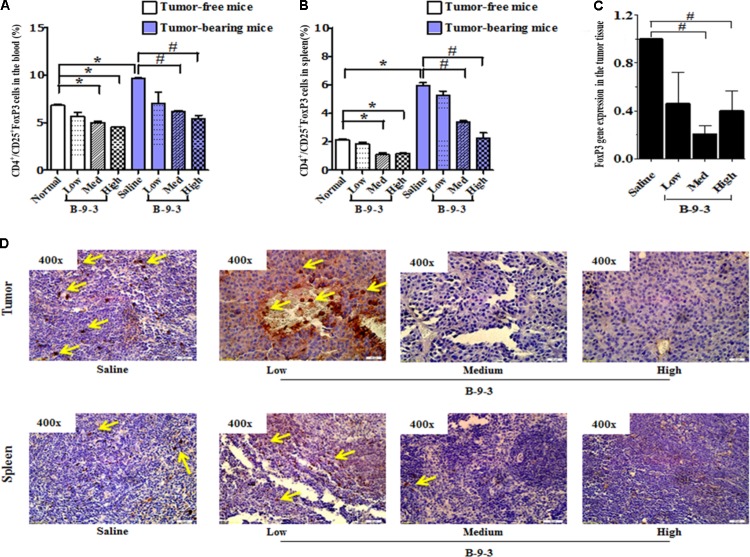
Effect of B-9-3 on CD4^+^/CD25^+^FoxP3 (Treg cells). **(A)** CD4^+^/CD25^+^FoxP3 cells in the blood; **(B)** CD4^+^/CD25^+^FoxP3 cells in spleen; **(C)** FoxP3 gene expression in the tumor tissue; **(D)** Immunohistochemistry of tumor and spleen for FoxP3 expression. C57BL mice were injected with 10^6^ cells in the right flank and mice were killed on day 22. Immunohistochemistry was performed following the protocol using monoclonal anti-FoxP3 antibody. “^∗^” versus normal group and “^#^” versus saline group; ^∗^ or ^#^ if *p* < 0.05 and ^∗∗^ or ^##^ if *p* < 0.01.

**FIGURE 5:**
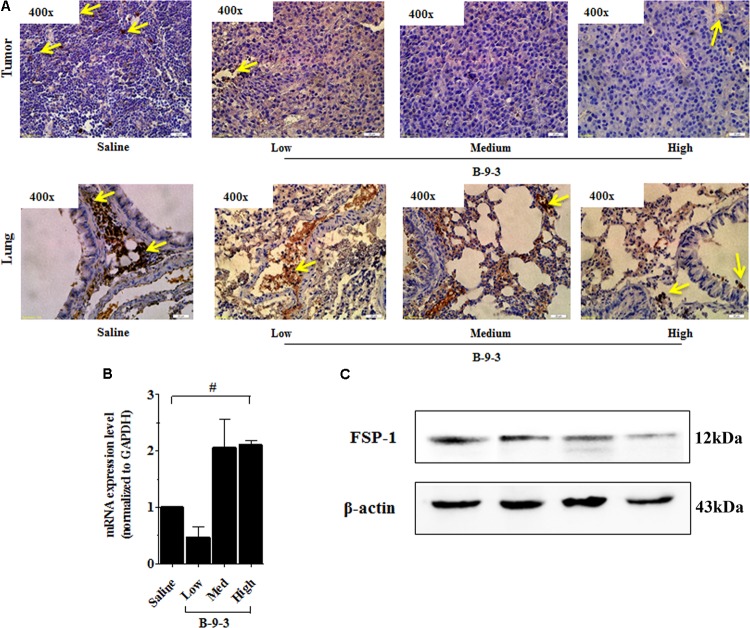
Effect of B-9-3 on tumor fibroblasts. **(A)** Immunohistochemistry of tumor and lung for FSP-1, **(B)** qPCR of FSP-1 in the tumor, **(C)** Western blot of FSP-1 protein in the tumor. C57BL mice were injected with 10^6^ cells in the right flank and mice were killed on day 22. Immunohistochemistry was performed following the protocol using a monoclonal anti-FSP1 antibody. “^#^” versus saline group; ^#^ if *p* < 0.05, ^##^ if *p* < 0.01 and ^###^ if *p* < 0.001.

**FIGURE 6:**
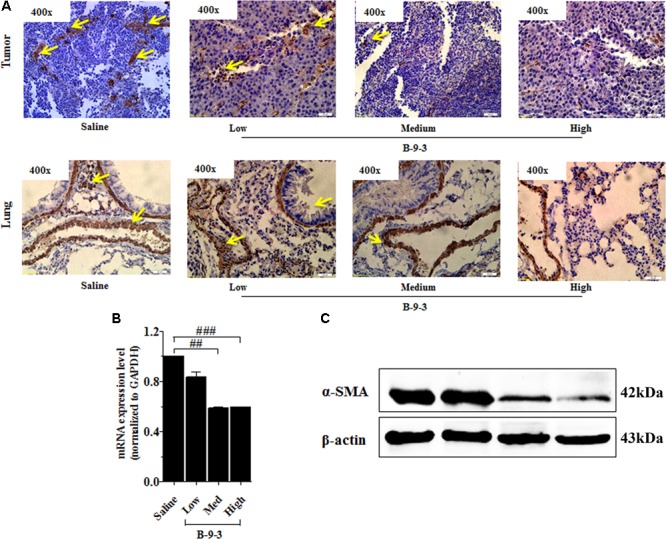
Effect of B-9-3 on tumor myofibroblasts. **(A)** Immunohistochemistry of tumor and lung for α-SMA; **(B)** qPCR of α-SMA in the tumor; **(C)** Western blot of α-SMA protein in the tumor. C57BL mice were injected with 10^6^ cells in the right flank and mice were killed on day 22. Immunohistochemistry was performed following the protocol using a monoclonal anti-α-SMA antibody. “^#^” versus saline group; ^#^ if *p* < 0.05, ^##^ if *p* < 0.01 and ^###^ if *p* < 0.001.

**FIGURE 7:**
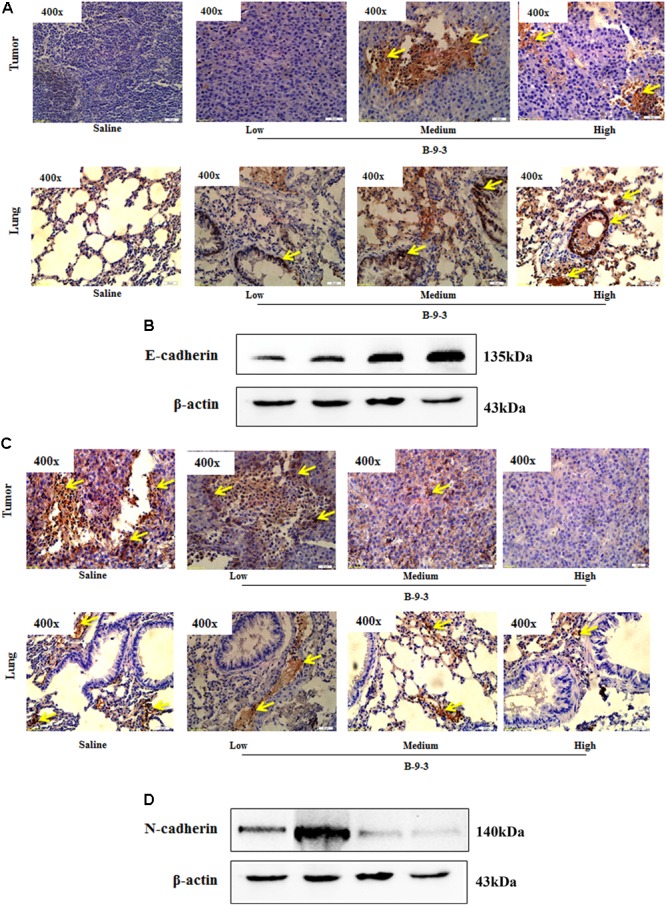
Effect of B-9-3 on epithelial-to-mesenchymal transition (EMT). **(A)** Immunohistochemistry of tumor and lung for E-cadherin; **(B)** Western blot of E-cadherin protein in the tumor. C57BL mice were injected with 10^6^ cells in the right flank and mice were killed on day 22. Immunohistochemistry was performed following the protocol using monoclonal anti-E-cadherin and anti-*N*-cadherin antibodies. **(C)** Immunohistochemistry of tumor and lung for *N*-cadherin; **(D)** Western blot of *N*-cadherin protein in the tumor. C57BL mice were injected with 10^6^ cells and mice were killed on day 22. Immunohistochemistry was performed following the protocol using monoclonal anti-E-cadherin and anti-*N*-cadherin antibodies.

**FIGURE 8:**
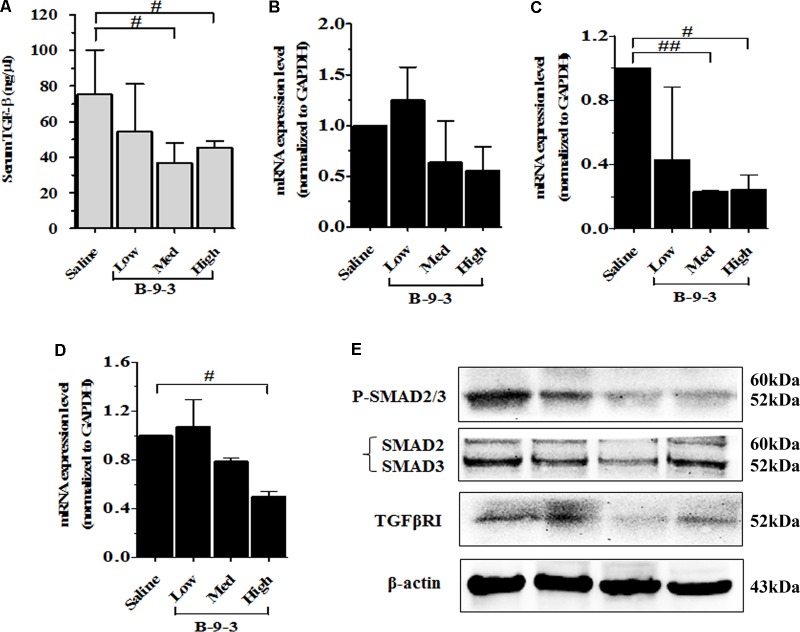
The implication of TGF-β signaling pathway in B-9-3 activity. **(A)** ELISA analysis for serum TGF-β1; **(B), (C)**, and **(D)** qPCR analysis for mRNA expression of TGFβRI, TGFβRII, and TGFβRIII, respectively; **(E)** Western blot analysis for β-actin, TGF-β receptor 1 (TGFβRI), total SMAD2/3 and phospho-SMAD2/3 complex. C57BL mice were injected with 10^6^ cells in the right flank and mice were killed on day 22. “^#^” versus saline group; ^#^ if *p* < 0.05, ^##^ if *p* < 0.01 and ^###^ if *p* < 0.001.

**FIGURE 9:**
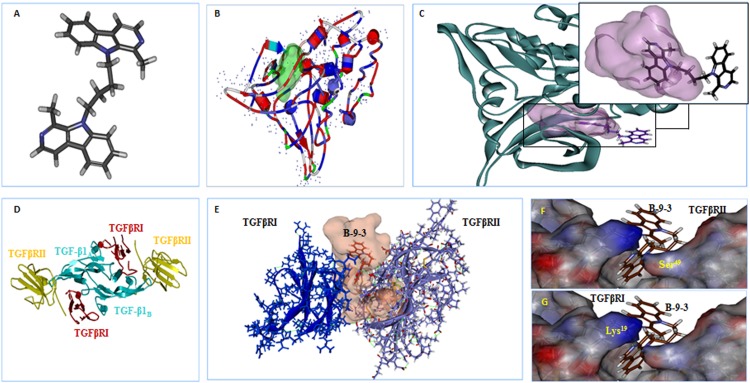
The *in silico* docking of B-9-3 and TGF-β receptors. **(A)** 3D Structure of B-9-3, dark gray are carbon atoms, blue are nitrogen atoms, and light gray are hydrogen atoms; **(B)** The Ribbon structure of TGFβ RI with the ATP-binding pocket of the kinase (Light green surface); **(C)** B-9-3 interaction with the kinase domain and posing of the small molecule inside the binding site pocket; **(D)** Ribbon drawing of the TGF-β1 ternary complex. TGF-β1 monomers (TGF-β1A and TGF-β1B) are colored cyan, TGFβR TGFβRII and I are colored red and yellow, respectively; **(E)** The interaction between of B-9-3 and the TGFβRI-TGFβRII complex and posing of the small molecule between the TGFβRI and TGFβRII; **(F)** The hydrogen bond between B-9-3 and the Ser49of TGFβRII (distance = 1.110 Å); **(G)** The hydrogen bond between B-9-3 and the Lys19o (distance = 1.006 Å) of TGFβRI. All figures and all subsequent ribbon drawings are prepared using Accelrys Discovery studio molecular graphics system.

Recently, growing evidence indicates that CD4^+^CD25^+^FOXP3^+^ regulatory T cells (Tregs) play a significant role in the control of tumor immunity, immune evasion and therapy resistance (Mandal et al., 2005; Bhattacharyya et al., 2010). In this study, B-9-3 in both standard and tumor-bearing mice reduced the number of CD4^+^CD25^+^FOXP3^+^ regulatory T cells in both the blood and in the spleen (**Figures 4A,B**). Also, the expression of FoxP3 at the mRNA level in the tumor tissue was significantly reduced in the groups of medium and high doses of B-9-3 (**Figure 4C**). To confirm the effect of B-9-3 on Treg population, we performed immunohistochemistry analysis of tumor tissue and spleen using a monoclonal antibody against Foxp3. As depicted in **Figure 4D**, B-9-3 significantly reduced the number of regulatory cells presenting FOXP3 marker in a dose-dependent manner. The increase of both CD4^+^ and CD8^+^ T cells along with the reduction of Tregs in tumor-bearing host confirmed the immune stimulation exerted by B-9-3.

### The Effect of B-9-3 on Cancer-Associated Fibroblasts (CAFs) in the Tumor Microenvironment (TME)

B-9-3 showed an immunomodulatory robust impact not only in the circulatory and splenic compartments but also in the TME as reflected by the elevation of CD4^+^ and CD8^+^ T cells and the decrease of immunosuppressive Tregs (Ha, 2009). Hence, we investigated the effect of this drug on other immunosuppressive cells as well.

Therefore, the effect of B-9-3 on the TME was investigated by exploring CAFs. CAFs include both fibroblasts expressing FSP-1 and myofibroblasts with their marker α-SMA. Here, the expression of FSP-1 and α-SMA was assessed in the primary tumor and also the lung, which is the familiar site of metastases from LLC cells (Genin et al., 2008). The presence of activated myofibroblasts expressing α-SMA was detected in pulmonary metastases of certain tumors (Gravdal et al., 2007; Michalik et al., 2011). In this study, immunohistochemistry and western blot analyses shown in **Figure 5** revealed a low level of the fibroblast marker in both the TME and lung. On the contrary, the gene expression of FSP-1 was up-regulated in the tumor. Additionally, the myofibroblasts marker α-SMA was down-expressed at both the gene level, as showed qPCR results, and the protein level which was confirmed by western blot and immunohistochemistry analyses (**Figure 6**).

### The Effect of B-9-3 on Epithelial to Mesenchymal Transition (EMT) and TGF-β Signaling Pathway *in Vivo*

Since B-9-3 reduced the expression of α-SMA in both primary tumor and lung; we thought it might inhibit the EMT, a phenomenon that contributes to an increased tumor cell motility and invasive behavior. EMT is generally characterized by reduced E-cadherin and increased *N*-cadherin expression (Monsef et al., 2004). **Figure 7** shows the effect of B-9-3 on both markers in the tumor tissue and lung. As demonstrated in the figure, B-9-3 increased the level E-cadherin and mitigated that of *N*-cadherin in a dose-dependent manner.

Because TGF-β is a critical growth factor that plays a crucial role in the TME, including the regulation and the expansion of regulatory T cells (Treg), induction of myofibroblasts from fibroblasts and the regulation of EMT (Kojima et al., 2010; Bellomo et al., 2016), we hypothesized that B-9-3 effect in the TME may be due to the modulation of TGF-β signaling pathway. To investigate the involvement of this pathway in B-9-3 activity we first performed ELISA analysis on animal serum. **Figure 8A** showed that B-9-3 significantly decreased the level of TGF-β1 in the blood, at medium and high doses. Further, qPCR was conducted to evaluate the gene expression of the three TGF-β receptors including TGFβRI, TGFβRII, and TGFβRIII. **Figures 8B–D** confirm that only high dose of B-9-3 decreased the gene expression of TGFβRII and TGFβRIII. On the contrary, the low dose did not affect. Moreover, B-9-3 at a medium dose reduced only TGFβRII gene expression in a significant way. The effect of B-9-3 on TGFβRI was not remarkable neither on the mRNA nor the protein level (**Figures 8B,E**). The activation of TGF-β receptors I and II leads to a signal that is transmitted inside the cell by SMAD proteins after their phosphorylation. If it was true, that B-9-3 can down-regulate TGFβRII, then SMAD phosphorylation must be interrupted. As displayed in **Figure 8E**, B-9-3 at medium and high doses significantly decreased the levels of both phosphorylated SMAD2 and 3 which confirms the previous results indicating the modulation of TGF-β signaling pathway by this drug.

### The *in Silico* Docking of B-9-3 and TGF-β Receptors

Because B-9-3 modulated TGF-β signaling pathway *in vivo*, we explored the possibility of interactions between B-9-3 and TGF-β receptors I and II and discussed the nature of these interactions. In this step, we conducted docking studies using CDOCKER from the Discovery Studio 2.5 software package to investigate any possible interaction between B-9-3 and TGF-β receptors.

The results showed that B-9-3 could interact with the kinase domain of TGFβRI within the hydrophobic ATP-binding pocket due to hydrophobic interactions (**Figures 9A,B**); no hydrogen bonds were detected between the drug and different amino acids from the binding site (**Figure 9C**). We further explored the possible interaction of B-9-3 with the ternary complex formed of TGF-β1 and the extracellular domains of TGFβRI and TGFβRII (**Figures 9D,E**). This complex is necessary to induce proximity productive conformation for the intracellular serine/threonine kinase domain of the receptors, which facilitates the phosphorylation. Interestingly, the computer-aided simulation results showed that B-9-3 could interact with Lys19 of TGFβRI and the Ser49 of the TGFβRII (**Figures 9F,G**). These results may indicate that B-9-3 can simultaneously perturb the binding of TGF-β1 with the TGFβRI via interaction with Lys19 and the complex TGFβRII- TGFβRI through binding with Ser49.

#### The Effect of B-9-3 on Fibroblast to Myofibroblast Transition

Besides molecular simulation studies, we also performed a FMT model by treating fibroblasts with human recombinant TGF-β1 following a protocol that has already been reported (Akhurst and Hata, 2012). Myofibroblasts constitute the leading members of CAFs in the TME that play crucial roles in tumor development and metastasis. Since myofibroblasts formation is under control of TGF-β signaling pathway, we study FMT as a model for the modulation of TGF-β pathway by B-9-3 *in vitro* with emphasis on the role of TGF-β receptors in this effect.

**FIGURE 10:**
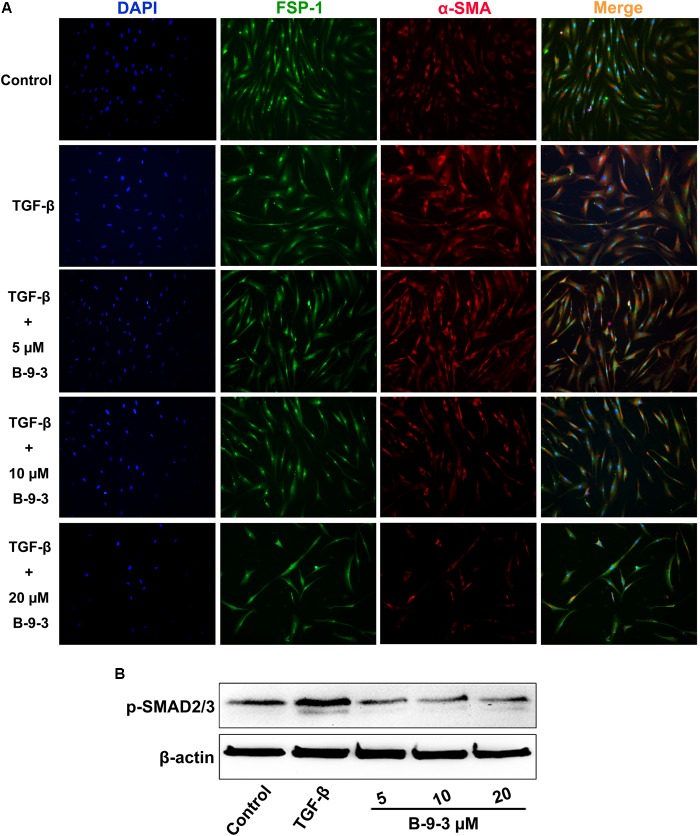
The *in vitro* effect of B-9-3 on fibroblast-to-myofibroblast transition (FMT). Human fibroblasts were cultured in DMEM supplemented with 10% (v/v) fetal bovine serum at 37°C and 5% CO2. Cells were treated with different concentrations of B-9-3 for 1 h before 5 ng/ml of TGF-β was added, and cells were further cultured for 72 h. Immunofluorescence and western blot were performed as previously described. **(A)** Immunofluorescence of human fibroblasts for FSP-1 and α-SMA; **(B)** Western blot analysis for phosphorylated SMAD (P-SMAD), 200×.

TGF-β is capable of inducing the transition of fibroblasts (FSP-1) to myofibroblasts (α-SMA). In our investigation, only 5 μM of B-9-3 could block the FMT in a TGF-β-dependent way as revealed by the decrease of the phosphorylation of SMAD2/3 (**Figures 10A,B**). Further concentrations (10 and 20 μM) were toxic and therefore were not used in the present investigation.

#### The Implication of TGF-β Pathway in B-9-3’s Activity *in Vitro* on Fibroblasts

**FIGURE 11:**
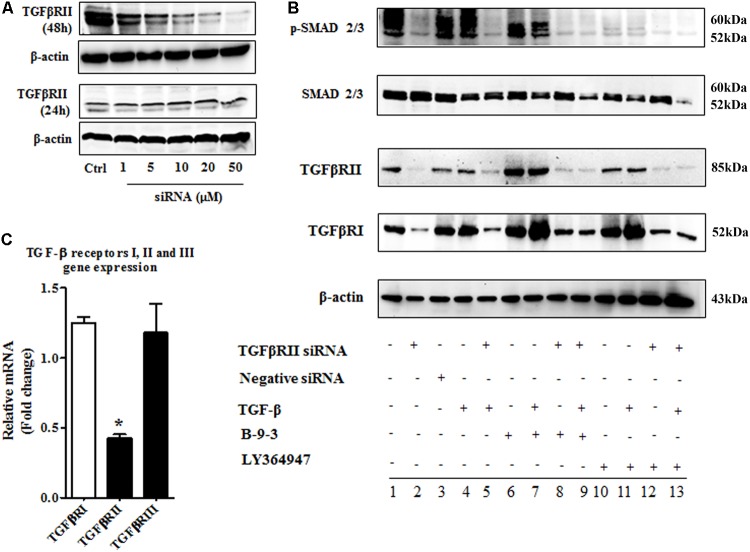
The implication of TGF-β receptor 2 (TGFβRII) in the effect of B-9-3 on FMT. **(A)** Determination of the best transfection conditions; human fibroblasts were cultured in DMEM supplemented with 10% FBS without antibiotics for 48 h then cells were treated with different concentrations of TGFβRII siRNA as described above for 24 or 48 h; western blot analysis for TGFβRII protein was performed as previously described; **(B)** Evaluation of the specificity of TGFβRII siRNA; fibroblasts were cultured in DMEM supplemented with 10% FBS without antibiotics for 48 h then cells were treated with 10 μM of TGFβRII siRNA as described above for 48 h; qPCR was performed on total RNA of fibroblasts treated with different drugs for 24 h; **(C)** Effect of B-9-3 on TGF-β signaling pathway elements; fibroblasts were cultured in DMEM supplemented with 10% FBS without antibiotics for 48 h then cells were treated with 10 μM of TGFβRII siRNA as described above for 48 h; western blot analysis for was performed on total protein of fibroblasts treated with different drugs for 24 h.

After confirming the effect of B-9-3 on FMT and TGF-β signaling pathway *in vitro*, we explored the role of TGF-β receptors I and II in this effect. We adopted two commonly used methods to inhibit TGF-β receptors I and II. First, LY364947: a small molecule inhibitor of TGFβRI, TGFβRII with IC50 values of 0.059 and 0.4 μM, respectively; second, a small interfering RNA (siRNA) for TGF-β receptor II (with 2 RefSeq (NM_001024847.2 and NM_003242.50) which was validated in cell-based tests by the provider. From the first step, the best transfection conditions of fibroblasts were determined as shown in **Figure 11A**; 48 h of transfection had a better outcome, and 10 μM decreased TGFβRII protein by more than 60% therefore for following studies, fibroblasts were treated with 10 μM of TGFβRII siRNA for 48 h. The specificity of this siRNA was also tested as shown in **Figure 11B**.

Also, fibroblasts were silenced using TGFβRII siRNA then treated with either B-9-3 or LY364947 in the presence or absence of the ligand TGF-β giving a total of 13 groups as shown in **Figure 11C**. First, the siRNA significantly reduced the level of TGFβRII protein, but also decrease slightly that of TGFβRI which might be due to the lack of high specificity of anti-TGFβRI antibody since qPCR results did not show a down-regulation of this receptor at the gene level. A negative control siRNA was employed and showed no influence on both receptors (group 3). It is worth mentioning that the presence of the ligand, TGF-β, did not affect the silencing effect on both receptors (group 4).

Surprisingly both B-9-3 and LY364947 increased the level of TGFβRI significantly in non-silenced cells only (groups 6, 7, 10, and 11). The increase of TGFβRI protein was slightly higher in cells pre-incubated with TGF-β prior treatment with the drugs (groups 7 and 11). It might indicate that the presence of the ligand increases the expression of TGFβRI via a positive feedback mechanism. Silencing of TGFβRII lessened the effect of both drugs on TGFβRI protein level (groups 8, 9, 12, and 13).

The action of both drugs on TGFβRII protein level was also evaluated. In non-silenced cells (groups 6, 7, 10, and 11), B-9-3 and LY364947 did not cause a significant change of TGFβRII level, and the presence or absence of the ligand TGF-β was not impactful. It might signify that the lack of any positive feedback by the ligand on the expression of TGFβRII. Further, B-9-3 and LY364947 did not affect TGFβRII level in silenced cells (groups 8, 9, 12, and 13).

The modulation of TGF-β signaling pathway was further explored by assessing the protein level of SMADs. First, non-silenced cells treated with only the ligand had a modest decrease of total SMAD2/3 protein which may suggest negative feedback due to excess of the signal. Whole SMADs level was not affected by B-9-3, and the only slight decrease was observed in silenced cells treated with this drug in the presence of TGF-β. Non-silenced fibroblasts are receiving LY364947, after pre-treated with the ligand, slightly reduced the level of total SMAD2/3 while silenced counterparts significantly reduced these proteins. Finally, the phosphorylated part of SMAD2/3, which is responsible for transmitting TGF-β signal into the nucleus, was evaluated using western blot. As shown in **Figure 11C**, silencing of TGFβRII significantly reduced the phosphorylation of SMAD2/3, and the presence or absence of the ligand did not change this effect (group 2 and 5, respectively). Moreover, B-9-3 inhibited the phosphorylation of SMAD2/3 in a significant way. Finally, the result of LY364947 on p-SMAD2/3 was stronger as shown in **Figure 11C**.

## Discussion

Lung cancer is one of the leading causes of cancer-related death worldwide. In our continuous search for new plant-based antitumor agents, alkaloids from Peganum harmala *L* appeared up-and-coming candidates because of their potent cytotoxicity against tumor cells. Harmala alkaloids can serve for the design and rational planning of new agents with potential therapeutic properties and minimal side effects (Ogunjimi et al., 2012).

The principal constituents of these alkaloids are β-carbolines, such as harmane. In the present work, we explored the antitumor effect of a harmane derivative B-9-3 which is formed of two harmane rings linked by a butyl group. In a first step, we investigated the anti-tumor effect of B-9-3 in a mouse orthotopic model of human cell line A549 in nude mice and a mouse LLC model in C57BL/6 immunocompetent mice. Because our preliminary data (not shown) also suspected B-9-3 to modulate the immune system function, we aimed to verify that using these two different models. A549 cell line is an NSCLC cell line which is widely utilized to simulate human lung cancers in female BALB/c nude mice. On the contrary, LLC cells were initially established from male C57BL mice and are widely used to explore lung metastases and mechanism of action of multiple anti-cancer agents. The data obtained from the human A549 transplantation in BALB/c nude mice did not reveal any significant decrease of the animals’ body weight at the high dose. Meanwhile, the drug antitumor effect was moderate at both medium (32% inhibition) and high dose (30.2% inhibition). Although B-9-3 significantly decreased the tumor growth in A549 xerographs, its effect was not so strong which led us to study the activity of B-9-3 in immunocompetent mice so check whether the presence of the immune system may increase the anti-tumor potency. As revealed in our investigation, B-9-3 significantly reduced tumor volume during the study with a final inhibition rate of 46.9% at a medium dose and 47.5% at the high dose.

Because the anti-tumor activity of B-9-3 in immunocompetent mice was higher than that in nude mice, we speculated that B-9-3 possess an immunomodulatory effect. Therefore, we measured the percentages of CD4^+^, CD8^+^ and T regulatory cells (Tregs) in both peripheral blood and spleens of normal and tumor-bearing mice treated with the drug. The gene expression of CD4 and CD8 markers were also evaluated in the tumor tissue. CD4^+^ and CD8^+^ T cells are the primary effectors against several different tumors, and their increase in the peripheral blood and spleen may reflect an immunostimulatory and an enhancement of their bioavailability in the tumor site. T cells in tumors—the so-called tumor infiltrating lymphocytes (TIL), which include both CD4^+^ and CD8^+^ T cells, have compelling clinical relevance as a critical denominator for OS in cancer patients (Anastasiou, 2017). Our results showed that B-9-3, in both standard and cancer mice, enhanced the level of CD4^+^ and CD8^+^ T cells in the circulatory and splenic compartments besides the primary tumor site. Additionally, we measured the number of circulatory and splenic and T regulatory cells and found lower percentage in mice treated with B-9-3 especially at medium and high doses; T regulatory cells were also depleted in the tumor tissue of treated mice. These results may justify the higher antitumor effect of this drug in immunocompetent mice.

The modulation of TME by B-9-3 was further investigated by exploring its activity on CAFs. CAFs include fibroblast expressing FSP-1 and myofibroblasts or activated fibroblasts expressing α-SMA which are often observed in the stroma of various human carcinomas where their presence in large numbers is associated with higher-grade malignancy and poor prognosis. The expression of these two markers was evaluated in the primary tumor and the lung to detect any metastases from LLC cells. Recently, myofibroblasts expressing α-SMA were found in pulmonary metastases from certain types of cancer (Franco et al., 2010). The generation of these cells is under the control of TGFβ that is highly available in the tumor site and which induces fibroblast to become myofibroblasts. Fibroblasts are a type of cells that produce the extracellular matrix (ECM) and collagen and express FSP-1. In our investigation, the β-carboline “B-9-3” reduced the number of myofibroblasts in both the tumor tissue and lungs. The fibroblastic marker was down-regulated at the protein level but down-regulated at the mRNA level.

Then, we explored the effect of B-9-3 on the EMT, a phenomenon that contributes to an increased tumor cell motility and the invasive behavior. EMT is usually accompanied by a decrease of E-cadherin and an elevation of *N*-cadherin expression (Yoshida et al., 2014). B-9-3 increased E-cadherin and reduced *N*-cadherin in a dose-dependent manner in both the tumor and lungs.

The TME is highly immunosuppressive due to different secreted factors such as transforming growth factor 1 (TGF-β1). Because this cytokine is a critical growth factor that plays multiple roles in the TME including the regulation and the expansion of Treg cells, FMT and EMT, we hypothesized that B-9-3 anti-immunosuppressive effect was mediated via targeting TGF-β signaling pathway. As demonstrated from ELISA results, B-9-3 significantly decreased the level of TGF-β1 in the blood, at medium and high doses. Also, B-9-3 remarkably down-regulated TGFβRII and reduced the level of phosphorylated SMAD2/3 in the tumor tissue.

Next, we performed *in silico* docking studies and investigated whether B-9-3 can bind any of the TGF-β receptors. In general, TGF-β displays a high affinity only for type II receptors and does not frequently interact with isolated type I receptor (Callahan et al., 2002). Binding to the extracellular domains of TGFβR TGFβRII and I induce proximity and a productive conformation for the intracellular serine/threonine kinase domains of the receptors, facilitating the phosphorylation (Radaev et al., 2010) Upon phosphorylation TGF-β type I receptors phosphorylate down-stream effectors, the receptor-activated SMADs (R-SMADs) which assemble with SMAD4 and accumulate in the nucleus to regulate transcription via interaction with transcription factor partners; therefore, small molecule inhibitors of TGF-β signaling that specifically target the kinase domain of TGF-β type I receptor (TGFβRI), responsible for SMADs’ activation, have great therapeutic potentials especially in cancer (Wójcik et al., 2013). The first inhibitor developed to inhibit the TβRI kinase domain was a 2,4,5-substituted imidazole. Later, new 2,4,5-substituted pyrazole molecules were shown to target and strongly inhibit the TβRI ATP-binding pocket (Tojo et al., 2005). Recently, Eli Lilly started a phase Ib/2 clinical trial for pancreatic cancer patients with Gemcitabine and LY2157299, an imidazole derivative inhibitor of TβRI ATP-binding pocket (ClinicalTrials.gov identifier: NCT01373164). However, reports showed that these small molecules inhibitors might not be specific to TGFβRI. The lack of high specificity may be explained by the inherent structural analogy of the ATP-binding pocket of different kinases. Therefore, such compounds should be carefully monitored in human clinical trials, given some instances of drug resistance or some side effects such as cardiac toxicity reported in the literature (José, 2011).

In our investigation, B-9-3’s effect was more probably due to its interaction with the ternary complex formed of TGF-β1 and the extracellular domains of TGFβRI and TGFβRII. This complex, first reported by Radaev et al. (2010), was said to facilitate the phosphorylation by inducing proximity productive conformation for the intracellular serine/threonine kinase domain of the receptors. The complex was proved to require several amino acids’ intervention, from both the ligand and its two receptors, to achieve its function. On the one hand, the interaction between TGFβRI and the two monomers of TGF-β1 generates two hydrophobic patches. The first interface between consists of Trp30, Trp32, Tyr90, and Leu101 of the “palm” side of TGF-β1A fingers and Ile54, Pro55, and Phe60 from TGFβRI. The second patch consists of Ala1, Leu2, Asn5, and Tyr6 from α1-helix and Ile51, Gln57, and Lys60 from α3-helix of TGF-β1B contacting His15, Leu16, Lys19, Phe31, Ile54, and Val61 from TGFβRI with one hydrogen bond between the side chain of Tyr6 of TGF-β1 and His15 of TGFβRI. On the other hand, the interactions between TGF-β1 and TβRII involve five TGF-β1 residues (Arg25, His34, Tyr91, Gly93, and Arg94) at the tips of its fingers and seven TβRII residues (Phe30, Asp32, Ser49, Ile50, Ser52, Ile53, and Glu119) on the base of the toxin-fold fingers of the receptor. B-9-3 was found to interact with Lys19 of TGFβRI and the Ser49 of the TGFβRII which might be the mechanism by which this small molecule simultaneously perturbs the binding of TGF-β1 with TGFβRI and the complex TGFβRII- TGFβRI.

To confirm the implication of TGF-β signaling pathway in B-9-3’s effect *in vitro*, we investigated the impact of this small molecule on FMT. Because myofibroblasts are mainly induced in the TME by TGF-β signaling pathway, we studied FMT as a model for the modulation of this pathway by B-9-3. Our drug could block the FMT by decreasing the phosphorylation of SMAD2/3 complex. Furthermore, the role of TGF-β receptors I and II (TGFβRI and TGFβRII) in this effect was also examined. Therefore, we use gene silencing technique to knockdown TGFβRII in fibroblasts. We also used a small inhibitor LY364947 which is known to block the ATP-binding domain of both TGFβRI and TGFβRII. Our results indicate that, although B-9-3 slightly increased the protein level of the two receptors, it still blocked the phosphorylation of SMAD2/3 complex. It suggests that the effect of B-9-3 is most likely to be due to a molecular interaction with the receptors rather than decreasing their expression. It is worth mentioning that besides the striking effect of LY364947 on the phosphorylation of SMAD2/3 it also reduced the level of total SMAD2/3.

In summary, our investigation unveiled the potential antitumor and immunomodulatory activities of the new β-carboline derivative B-9-3 through the modulation of TGF-β. The *in silico* study revealed that B-9-3’s effect was more probably due to its interaction with the ternary complex rather than decreasing the expression of TGF-β receptors or interfering with their ATP-binding domains which may be a novel mechanism by which small molecules can inhibit the TGF-β signaling pathway without necessarily interacting with its ATP-binding domain. It also provided new insights into designing the active antitumor molecules on the TGF-β signaling pathway.

## Author Contributions

JS and JZ supervised the whole project. HZ and AD performed the major research and wrote the manuscript in equal contribution. JH, XA, CQ, LD, and YW provided the technical support. ZC provided their professional expertise.

## Conflict of Interest Statement

Xinjiang Huashidan Pharmaceutical Research CO., LTD., had no role in study design, data collection and analysis, decision to publish, or preparation of the manuscript. The authors declare that the research was conducted in the absence of any commercial or financial relationships that could be construed as a potential conflict of interest. The reviewer WZ and handling Editor declared their shared affiliation.

## References

[B1] Akhurst R. J. Hata A. (2012). Targeting the TGFβ signaling pathway in disease. *Nat. Rev. Drug Discov.* 11 790–811. 23000686 10.1038/nrd3810 PMC3520610

[B2] Anastasiou D. (2017). Tumour microenvironment factors are shaping the cancer metabolism landscape. *Br. J. Cancer* 116 277–286. 10.1038/bjc.2016.412 28006817 PMC5294476

[B3] Beauchemin N. (2011). The colorectal tumor microenvironment: the next decade. *Cancer Microenviron.* 4 181–185. 10.1007/s12307-011-0074-7 21735168 PMC3170422

[B4] Bellomo C. Caja L. Moustakas A. (2016). Transforming growth factor β as a regulator of cancer stemness and metastasis. *Br. J. Cancer* 115 761–769. 10.1038/bjc.2016.255 27537386 PMC5046208

[B5] Bertram J. S. Janik P. (1980). Establishment of a cloned line of Lewis Lung Carcinoma cells adapted to cell culture. *Cancer Lett.* 11 63–73. 7226139 10.1016/0304-3835(80)90130-5

[B6] Bhattacharyya S. Md Sakib Hossain D. Mohanty S. Sankar Sen G. Chattopadhyay S. Banerjee S. (2010). Curcumin reverses T cell-mediated adaptive immune dysfunctions in tumor-bearing hosts. *Cell. Mol. Immunol.* 7 306–315. 10.1038/cmi.2010.11 20305684 PMC4003225

[B7] Callahan J. F. Burgess J. L. Fornwald J. A. Gaster L. M. Harling J. D. Harrington F. P. (2002). Identification of novel inhibitors of the transforming growth factor beta1 (TGF-beta1) type 1 receptor (ALK5). *J. Med. Chem.* 45 999–1001. 11855979 10.1021/jm010493y

[B8] Calone I. Souchelnytskyi S. (2012). Inhibition of TGFβ signaling and its implications in anticancer treatments. *Exp. Oncol.* 34 9–16. 10.1158/1535-7163.MCT-12-1007 22453142

[B9] DuPage M. Dooley A. L. Jacks T. (2009). Conditional mouse lung cancer models using adenoviral or lentiviral delivery of CRE recombinase. *Nat. Protoc.* 4 1064–1072. 10.1038/nprot.2009.95 19561589 PMC2757265

[B10] Franco O. E. Shaw A. K. Strand D. W. Hayward S. W. (2010). Cancer-associated fibroblasts in cancer pathogenesis. *Semin. Cell Dev. Biol.* 21 33–39. 19896548 10.1016/j.semcdb.2009.10.010 PMC2823834

[B11] Genin O. Rechavi G. Nagler A. Ben-Itzhak O. Nazemi K. J. Pines M. (2008). Myofibroblasts in pulmonary and brain metastases of alveolar soft-part sarcoma: a novel target for treatment? *Neoplasia* 10 940–948. 18714394 10.1593/neo.08456 PMC2517638

[B12] Gravdal K. Halvorsen O. Haukaas S. A. Akslen L. A. (2007). A switch from E-cadherin to N-cadherin expression indicates epithelial to mesenchymal transition and is of strong and independent importance for the progress of prostate cancer. *Clin. Cancer Res.* 13 7003–7011. 18056176 10.1158/1078-0432.CCR-07-1263

[B13] Graves E. E. Maity A. Le Q. T. (2010). The tumor microenvironment in non-small cell lung cancer. *Semin. Radiat. Oncol.* 20 156–163. 10.1016/j.semradonc.2010.01.003 20685578 PMC2917385

[B14] Ha T. Y. (2009). The role of regulatory T cells in cancer. *Immune Netw.* 9 209–235. 20157609 10.4110/in.2009.9.6.209 PMC2816955

[B15] Hadrup S. Donia M. Thor Straten P. (2013). Effector CD4 and CD8 T cells and their role in the tumor microenvironment. *Cancer Microenviron.* 6 123–133. 10.1007/s12307-012-0127-6 23242673 PMC3717059

[B16] Han X. Zhang J. Guo L. Cao R. Li Y. Li N. (2012). A series of beta-carboline derivatives inhibit the kinase activity of PLKs. *PLoS One* 7:e46546. 10.1371/journal.pone.0046546 23056340 PMC3463587

[B17] Heinrich E. L. Walser T. C. Krysan K. Liclican E. L. Grant J. L. Rodriguez N. L. (2012). The inflammatory tumor microenvironment, epithelial-mesenchymal transition and lung carcinogenesis. *Cancer Microenviron.* 5 5–18. 10.1007/s12307-011-0089-0 21922183 PMC3343201

[B18] Isakov N. Feldman M. Segal S. (1984). Loss of the H-2.33 private specificity by 3LL tumor cells correlates with the tumor potential to metastasize across the H-2K region genetic barriers. *Exp. Clin. Immunogenet.* 1 170–174. 6401004

[B19] José M. G. (2011). *Signaling in the Heart.* New York, NY: Springer.

[B20] Kojima Y. Acar A. Eaton E. N. Mellody K. T. Scheel C. Ben-Porath I. (2010). Autocrine TGF-β and stromal cell-derived factor-1 (SDF-1) signaling drives the evolution of tumor-promoting mammary stromal myofibroblasts. *Proc. Natl. Acad. Sci. U.S.A.* 107 20009–20014. 10.1073/pnas.1013805107 21041659 PMC2993333

[B21] Kraus-Berthier L. Jan M. Guilbaud N. Naze M. Pierré A. Atassi G. (2000). Histology and sensitivity to anticancer drugs of two human non-small cell lung carcinoma implanted in the pleural cavity of nude mice. *Clin. Cancer Res.* 6 297–304. 10656461

[B22] Li Y. Liang F. Jiang W. Yu F. Cao R. Ma Q. (2007). DH334, a beta-carboline anti-cancer drug, inhibits the CDK activity of budding yeast. *Cancer Biol. Ther.* 6 1193–1199. 17622795

[B23] Mandal D. Bhattacharyya A. Lahiry L. Choudhuri T. Sa G. Das T. (2005). Failure in peripheral immuno-surveillance due to thymic atrophy: the importance of thymocyte maturation and apoptosis in adult tumor-bearer. *Life Sci.* 77 2703–2716. 16019036 10.1016/j.lfs.2005.05.038

[B24] Mathieu A. Remmelink M. D’Haene N. Penant S. Gaussian J. F. Van Ginckel R. (2004). Development of a chemoresistant orthotopic human nonsmall cell lung carcinoma model in nude mice: analyses of tumor heterogeneity in relation to the immunohistochemical levels of expression of cyclooxygenase-2, ornithine decarboxylase, lung-related resistance protein, prostaglandin E synthetase, and glutathione-S-transferase-alpha (GST)-alpha, GST-mu, and GST-pi. *Cancer* 101 1908–1918. 15386340 10.1002/cncr.20571

[B25] Michalik M. Pierzchalskab M. Włodarczyk A. Wójcik K. A. Czyż J. Sanak M. (2011). Transition of asthmatic bronchial fibroblasts to myofibroblasts is inhibited by cell-cell contacts. *Respir. Med.* 105 1467–1475. 10.1016/j.rmed.2011.04.009 21802932

[B26] Monsef H. R. Ghobadi A. Iranshahi M. Abdollahi M. (2004). Antinociceptive effects of *Peganum harmala* L. alkaloid extract on mouse formalin test. *J. Pharm. Pharm. Sci.* 7 65–69. 15144736

[B27] Ogunjimi A. A. Zeqiraj E. Ceccarelli D. F. Sicheri F. Wrana J. L. David L. (2012). Structural basis for specificity of TGFβ family receptor small molecule inhibitors. *Cell. Signal.* 24 476–483. 10.1016/j.cellsig.2011.09.027 21983015 PMC4490768

[B28] Palena C. Hamilton D. H. Fernando R. I. (2012). Influence of IL-8 on the epithelial-mesenchymal transition and the tumor microenvironment. *Future Oncol.* 8 713–722. 10.2217/fon.12.59 22764769 PMC3462442

[B29] Place A. E. Jin Huh S. Polyak K. (2011). The microenvironment in breast cancer progression: biology and implications for treatment. *Breast Cancer Res.* 13:227. 10.1186/bcr2912 22078026 PMC3326543

[B30] Radaev S. Zou Z. Huang T. Lafer E. M. Hinck A. P. Sun P. D. (2010). Ternary complex of transforming growth factor-β1 reveals isoform-specific ligand recognition and receptor recruitment in the superfamily. *J. Biol. Chem.* 285 14806–14814. 20207738 10.1074/jbc.M109.079921 PMC2863181

[B31] Rita M. Young I. (2004). Tumor skewing of CD34 + progenitor cell differentiation into endothelial cells. *Int. J. Cancer* 109 519–524. 10.1002/ijc.20003 14991572

[B32] Shi B. Cao R. Fan W. Guo L. Ma Q. Chen X. (2013). Design, synthesis and in vitro and in vivo antitumor activities of novel bivalent β-carbolines. *Eur. J. Med. Chem.* 60 10–22. 10.1016/j.ejmech.2012.11.033 23279863

[B33] Tojo M. Hamashima Y. Hanyu A. Kajimoto T. Saitoh M. Miyazono K. (2005). The ALK-5 inhibitor A-83-01 inhibits Smad signaling and epithelial-to-mesenchymal transition by transforming growth factor-beta. *Cancer Sci.* 96 791–800. 16271073 10.1111/j.1349-7006.2005.00103.x PMC11159601

[B34] Wójcik K. A. Skoda M. Koczurkiewicz P. Sanak M. Czyż J. Michalik M. (2013). Apigenin inhibits TGF-β1 induced a fibroblast-to-myofibroblast transition in human lung fibroblast populations. *Pharmacol. Rep.* 65 164–172. 23563034 10.1016/s1734-1140(13)70974-5

[B35] World Health Organization (2012). *Cancer Fact Sheets.* Geneva: WHO.

[B36] Xie G. Ji A. Yuan Q. Jin Z. Yuan Y. Ren C. (2014). Tumour-initiating capacity is independent of epithelial-mesenchymal transition status in breast cancer cell lines. *Br. J. Cancer* 110 2514–2523. 10.1038/bjc.2014.153 24755887 PMC4021510

[B37] Yoshida T. Ozawa Y. Kimura T. Sato Y. Kuznetsov G. Xu S. (2014). Eribulin mesilate suppresses experimental metastasis of breast cancer cells by reversing phenotype from epithelial-mesenchymal transition (EMT) to mesenchymal-epithelial transition (MET) states. *Br. J. Cancer* 110 1497–1505. 10.1038/bjc.2014.80 24569463 PMC3960630

[B38] Yu Y. Xiao C. H. Tan L. D. Wang Q. S. Li X. Q. Feng Y. M. (2014). Cancer-associated fibroblasts induce epithelial-mesenchymal transition of breast cancer cells through paracrine TGF-β signaling. *Br. J. Cancer* 110 724–732. 10.1038/bjc.2013.768 24335925 PMC3915130

[B39] Zhang J. Liu J. (2013). Tumor stroma as targets for cancer therapy. *Pharmacol. Ther.* 137 200–215. 23064233 10.1016/j.pharmthera.2012.10.003 PMC3556178

